# Retraction Note: RUNX3-regulated circRNA METTL3 inhibits colorectal cancer proliferation and metastasis via miR-107/PER3 axis

**DOI:** 10.1038/s41419-024-06497-w

**Published:** 2024-02-07

**Authors:** Feng Zhang, Tao Su, Meifang Xiao

**Affiliations:** 1 Department of Cardiovascular Medicine, Xiangya Hospital, Central South University, 410008 Changsha, Hunan P.R. China; 2 National Clinical Research Center for Geriatric Disorders, Xiangya Hospital, Central South University, 410008 Changsha, Hunan P.R. China; 3 The Institute of Medical Sciences, Xiangya Hospital, Central South University, 410008 Changsha, Hunan P.R. China; 4 Department of Health Management Center, Xiangya Hospital, Central South University, 410008 Changsha, Hunan Province P.R. China

**Keywords:** Rectal cancer, Rectal cancer

Retraction to: *Cell Death and Disease* 10.1038/s41419-022-04750-8, published online 16 June 2022

The Editors-in-Chief retracted this article because of concerns regarding a number of figures presented in this work. These concerns call into question the article’s overall scientific soundness and its authorship. An investigation conducted after its publication discovered similarities between images in this work and images in [1].

The image showing a pair of lungs labelled HCT15 (circMETTL3) in Figure 3E and the image showing a pair of lungs labelled HONE1 (miR-106a-5p inhibitor) in Figure 3G in [1].

The image showing a pair of lungs labelled Caco2 (circMETTL3) in Figure 3E and the image showing a pair of lungs labelled HONE1 (shTRIM24) in Figure 4G in [1].

The Editors-in-Chief therefore no longer have confidence in the integrity of the research presented in this article. The authors did not respond to the correspondence from the publisher about this retraction.
